# Nitric Oxide in Parkinson’s Disease: The Potential Role of Dietary Nitrate in Enhancing Cognitive and Motor Health via the Nitrate–Nitrite–Nitric Oxide Pathway

**DOI:** 10.3390/nu17030393

**Published:** 2025-01-22

**Authors:** Gianluca Tripodi, Mauro Lombardo, Sercan Kerav, Gilda Aiello, Sara Baldelli

**Affiliations:** 1 Department for the Promotion of Human Science and Quality of Life, San Raffaele Open University, Via di Val Cannuta, 247, 00166 Rome, Italy; gianluca.tripodi@uniroma5.it (G.T.); mauro.lombardo@uniroma5.it (M.L.); gilda.aiello@uniroma5.it (G.A.); 2 Department of Molecular Biology and Genetics, Çanakkale Onsekiz Mart University, 17100 Çanakkale, Türkiye; sercankarav@comu.edu.tr; 3 IRCCS San Raffaele Roma, 00166 Rome, Italy

**Keywords:** nitric oxide, Parkinson’s disease, nitric oxide synthase, dietary nitrate

## Abstract

Background/Objectives: Parkinson’s disease (PD) is a progressive neurodegenerative disorder characterized by the loss of dopaminergic neurons in the *substantia nigra pars compacta*, leading to motor symptoms such as tremor, rigidity, and bradykinesia. The pathological hallmarks of PD include Lewy bodies and mechanisms like oxidative/nitrosative stress, chronic inflammation, and mitochondrial dysfunction. Nitric oxide (NO), produced by nitric oxide synthase (NOS) isoforms, plays a dual role in neuroprotection and neurodegeneration. Excessive NO production exacerbates neuroinflammation and oxidative/nitrosative damage, contributing to dopaminergic cell death. This review explores NO’s role in PD pathogenesis and investigates dietary nitrate as a therapeutic strategy to regulate NO levels. Methods: A literature review of studies addressing the role of NO in PD was conducted using major scientific databases, including PubMed, Scopus, and Web of Science, using keywords such as “nitric oxide”, “NOSs”, “Parkinson’s disease”, and “nitrate neuroprotection in PD”. Studies on nitrate metabolism via the nitrate–nitrite–NO pathway and its effects on PD hallmarks were analyzed. Studies regarding the role of nitrosamine formation in PD, which are mainly formed during the nitrification process of amines (nitrogen-containing compounds), often due to chemical reactions in the presence of nitrite or nitrate, were also examined. In particular, nitrate has been shown to induce oxidative stress, affect the mitochondrial function, and contribute to inflammatory phenomena in the brain, another factor closely related to the pathogenesis of PD. Results: Excessive NO production, particularly from iNOS and nNOS, was strongly associated with neuroinflammation and oxidative/nitrosative stress, amplifying neuronal damage in PD. Dietary nitrate was shown to enhance NO bioavailability through the nitrate–nitrite–NO pathway, mitigating inflammation and oxidative/nitrosative damage. Conclusions: Dysregulated NO production contributes significantly to PD progression via inflammatory and oxidative/nitrosative pathways. Dietary nitrate, by modulating NO levels, offers a promising therapeutic strategy to counteract these pathological mechanisms. Further clinical trials are warranted to establish its efficacy and optimize its use in PD management.

## 1. Introduction

Parkinson’s disease (PD) is a progressive neurodegenerative disorder that predominantly affects dopaminergic neurons in the *substantia nigra pars compacta*, a region crucial for motor control. The loss of these neurons leads to a reduction in dopamine levels in the striatum, resulting in the characteristic motor symptoms of PD, such as resting tremor, muscle rigidity, bradykinesia, and postural instability [1]. At the cellular level, PD is characterized by the presence of Lewy bodies, intracellular inclusions composed mainly of misfolded α-synuclein and other proteins associated with oxidative stress and neuroinflammation [2,3]. Despite decades of research, the precise mechanisms driving neurodegeneration in PD remain largely unclear. Inflammation in the (CNS) is mainly mediated by microglia, which switch from a protective to a neurotoxic state under pathological conditions [4]. This inflammatory environment perpetuates a chronic cycle of neurodegeneration, in which damaged cells release danger signals that further activate microglia [5]. Correspondingly, oxidative/nitrosative stress plays a fundamental role in PD, in which dopamine oxidation, generation of reactive oxygen species (ROS), reactive nitrogen species (RNS), and impaired antioxidant defenses contribute to neuronal damage. In addition, mitochondrial dysfunction, evidenced by reduced respiratory chain complex I activity, is another crucial element [6,7] both compromising ATP production and increasing ROS/RNS production, resulting in further exacerbation of oxidative damage [8].

A pivotal element in these processes is nitric oxide (NO), a signaling molecule involved in numerous physiological and pathological pathways. NO is a highly reactive gas that serves as a chemical messenger in many biological functions, including the control of vasodilation, modulation of the immune response, and regulation of synaptic plasticity [9]. In the CNS, NO is synthesized by three isoforms of nitric oxide synthase (NOS): neuronal (nNOS), endothelial (eNOS), and inducible (iNOS), each with a specific and distinctive role. nNOS is expressed mainly in neurons and plays a fundamental role in the modulation of synaptic transmission, regulating both communication between neurons and synaptic plasticity, a process essential for learning and memory [10]. eNOS is mainly involved in the vascular system, where it regulates vascular tone and blood flow, while iNOS is expressed in response to inflammatory and immune stimuli and is characterized by a very high and prolonged production of NO [11]. Excessive NO production, particularly when mediated by iNOS and nNOS, can have deleterious effects on the CNS. Excess NO, in fact, contributes to neuroinflammation, generating RNS and creating the conditions for oxidative stress. The massive production of NO mediated by iNOS is involved in glial inflammation, which further amplifies neuronal damage and promotes a pathological cycle that accelerates the degeneration of dopaminergic neurons in PD [12]. This imbalance between physiological and pathological NO production represents an important mechanism in the progression of PD, and therefore a potential therapeutic target to modulate neuroinflammation and improve neuronal survival.

As a precursor to NO [13], dietary nitrate has recently attracted increasing interest for its potential neuroprotective role. Indeed, it is believed that a greater intake of nitrate could both improve cardiovascular health and protect against neurodegeneration. Nitrate is found in many green leafy vegetables such as spinach, lettuce, and beets. When ingested, it is metabolized in the human body to nitrite, which in turn can be reduced to NO through a physiological cycle known as the ‘nitrate–nitrite–NO pathway’ [14]. This process involves the conversion of nitrate to nitrite mainly via bacterial nitrate reductases on the surface of the tongue. Although nitrate reduction also occurs in some body tissues (e.g., in the liver), the rate and extent are significantly lower than in the oral cavity. For example, the use of mouthwashes containing chlorhexidine, which interrupts oral nitrate reduction, generally inhibits nitrite formation by over 90% [15]. Several studies suggest that dietary nitrate supplementation may improve endothelial function, a key aspect of vascular health, through increased bioavailability of NO [16]. Additionally, dietary nitrate has been shown to modulate inflammation by reducing immune cell activation and lowering levels of inflammatory cytokines [17]. These anti-inflammatory effects, combined with the ability to reduce oxidative stress, offer potential benefits in the treatment of neurodegenerative diseases, including PD [18,19]. Clinical studies have suggested that regular intake of dietary nitrate through supplements or nitrate-rich foods might positively affect the cognitive function and neuronal protection of patients with neurodegenerative diseases such as PD, reducing neuronal damage and improving motor functions [20].

This review aims to explore the role of nitric oxide NOS in PD, highlighting the complex mechanisms through which it may contribute to neurodegeneration. Furthermore, the effects of dietary nitrate, a natural source of NO, will be examined, along with its potential as a therapeutic strategy to modulate NO levels in the brain, improving endothelial function and counteracting pathological processes related to Parkinson’s disease. Nitrate, through the nitrate–nitrite–NO pathway, represents a promising modality to balance the beneficial and detrimental effects of NO in the neurodegenerative context, opening new therapeutic possibilities for the treatment of PD.

## 2. Materials and Methods

The review was conducted following the Scale for the Assessment of Narrative Review Articles (SANRA). Searches were conducted on PubMed, Scopus, and Web of Science to ensure a comprehensive collection of studies related to the role of NO in Parkinson’s disease and potential therapeutic applications of dietary nitrate. Keywords such as ‘nitric oxide’, OR ‘nitric oxide synthase’, OR ‘neuroprotective’, AND ‘Parkinson’s disease’ were used, along with Boolean operators to optimize the search strategy.

### 2.1. Inclusion Criteria

The studies included in this work met the following criteria: (a) investigated the role of NO in the pathogenesis of PD or the therapeutic effects of dietary nitrate; (b) were clinical studies, preclinical studies, cohort studies, or meta-analyses; (c) were published in English between 1 January 1990 and 31 October 2024.

### 2.2. Exclusion Criteria

The studies excluded from this work met the following criteria: (a) were review articles, editorials, conference abstracts, or case reports; (b) did not report direct evidence linking NO or dietary nitrate to mechanisms or interventions on PD; (c) focused on irrelevant populations (e.g., pediatric or non-neurodegenerative diseases).

### 2.3. Data Extraction

**Figure 1:**
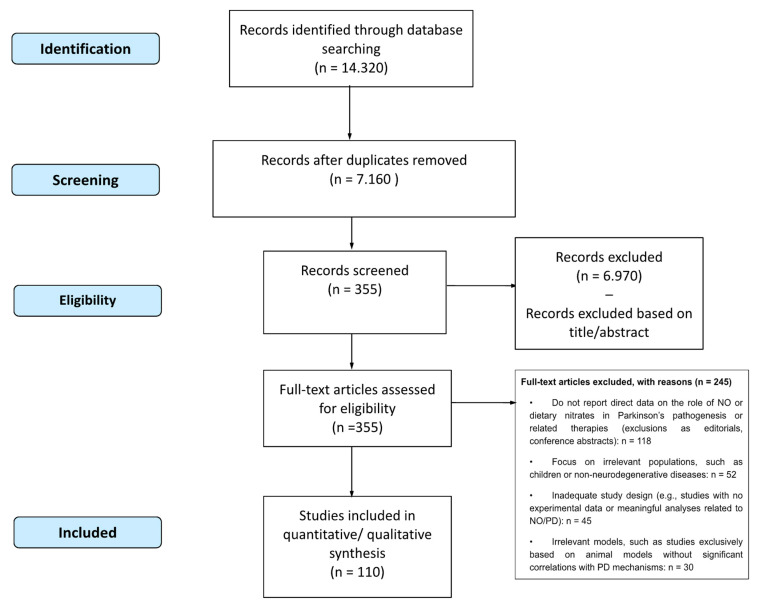
Flow diagram of the literature search and study selection process.

A flow chart (Figure 1) illustrates the selection process, from the initial database search to the final selection of studies to be included. Data were extracted by two independent reviewers (S.B. and G.A.), including study design, participant characteristics, intervention details and outcomes. Discrepancies were resolved by consulting a third reviewer (M.L.).

## 3. Role of NOSs in PD

NO biosynthesis by NOS enzymes requires L-arginine, NADPH, and oxygen to produce NO and L-citrulline in a reaction, which involves essential cofactors [21]. NOSs are enzymes that have been cloned and purified: nNOS, eNOS, and iNOS [22]. nNOS and eNOS are constitutively expressed and calcium (Ca^2+^)-dependent enzymes. nNOS is mainly expressed in cells of neuronal origin where NO functions as neuromodulator and neurotransmitter in the central and peripheral nervous system. Moreover, the nNOS isoform is also present at the skeletal and cardiac muscle level, namely the nNOSµ. eNOS is localized in the endothelium, in cardiomyocytes, and in adipocytes. It principally participates in the regulation of blood pressure and vascular tone. The activity of nNOS and eNOS, both of which are present in neuronal tissue all throughout the brain, is highly dependent on intracellular Ca^2+^ levels, especially nNOS, which is linked to N-methyl-d-aspartate receptors (NMDAR). Instead, iNOS is a Ca^2+^-independent and cytokine-inducible enzyme, expressed in macrophages during inflammation and tissue injury [23]. In recent years, the importance of the fourth isoform of NOS has emerged: the mitochondrial NOS (mtNOS). It is localized in the mitochondrial inner membrane of several tissues, including skeletal muscle, and could be of key importance in oxygen uptake and apoptosis through cytochrome *c* release [18,24]. Recent studies have shown that mtNOS has homology with nNOS and could be one of its splice variants [25,26]. Nevertheless, the precise nature of mtNOS is still under debate. Besides its pivotal role in regulating oxygen uptake and cellular signaling [25,27], mtNOS positively regulates respiratory function and the release of cytochrome *c* into the cytosol, a critical event for the initiation of apoptosis [28].

nNOS and iNOS appear significantly implicated in PD pathophysiology [7]. In post-mortem analyses, an increased concentration of nNOS was observed both in midbrain samples from PD patients and in PD model animals [29]. In weaver mice, characterized by a spontaneous depletion of dopaminergic neurons, an increase in nNOS expression was observed specifically in the *substantia nigra*, without involvement of other brain areas [30]. These findings suggest a potential role of nNOS activity in the pathogenesis of PD. nNOS activity is mainly regulated through post-translational modifications and interaction with specific proteins. For example, phosphorylation of the Ser847 residue inhibits enzymatic activity, while dephosphorylation of the same residue stimulates it [31]. In this context, docosahexaenoic acid, a compound able to phosphorylate nNOS and therefore reduce its activity, has been shown to be protective against 1-methyl-4-phenyl-1,2,3,6-tetrahydropyridine (MPTP)-induced toxicity in dopaminergic neurons in animal models of PD [32].

Among the proteins interacting with nNOS, HSP90 (heat shock protein 90) emerges as a crucial regulator both for its role in maintaining protein homeostasis and for its ability to prevent aggregation of the protein alpha-synuclein (aSyn), a key feature of PD [33]. Although this function suggests a potential neuroprotective role, HSP90 is also able to interact with nNOS, amplifying its activity and thus contributing to the generation of RNS. In cellular models of PD, inhibition of HSP90 has been shown to prevent pathological features of the disease, such as neurite loss and aSyn aggregation [34]. Finally, the reduction in nNOS expression by siRNA in cellular and animal models of PD has shown a protective effect on dopaminergic neurons [35]. A comprehensive analysis of the collected data supports the hypothesis that nNOS is upregulated in PD and that its inhibition may represent a promising strategy to counteract its progression.

Another study found that the iNOS gene was upregulated in the *substantia nigra* of PD patients [36]. This finding is further supported by studies showing increased iNOS expression in several animal models of PD, such as those based on 6-hydroxydopamine (6-OHDA), MPTP, and aSyn oligomers [37,38,39]. Furthermore, mice that genetically lack iNOS show increased resistance to stressors that normally induce PD-like symptoms [40]. Importantly, glial cells tend to accumulate in areas of the brain affected by active neurodegeneration, contributing to the production of high levels of NO, which can intensify toxicity to surrounding cells.

To date, the mechanism by which NOSs are activated in this pathology is still not entirely clear. Some authors think that it is the neuroinflammation, which typically characterizes PD patients, that activates the microglia and thus induces iNOS [41]. In particular, chronic inflammation is a key factor in the pathogenesis of PD, contributing to the neurodegeneration of dopaminergic neurons in the *substantia nigra*. A central element of this process is activated microglia, which serve as the first line of immune defense in the central nervous system. However, when overactivated, microglia release pro-inflammatory cytokines, such as IL-1β, TNF-α, and IFN-γ, creating a neurotoxic environment that further damages neurons and causes uncontrolled activation of iNOS with subsequent production of NO [42]. In PD patients, uncontrolled increase in NO has been linked to mitochondrial dysfunction, reduced ATP production, and activation of cell death mechanisms, such as apoptosis and necrosis [43]. It has also been shown that NO release, by amplifying oxidative stress, further activates microglia, thus perpetuating inflammation. This chronic cycle contributes to neuronal degeneration and accelerates the progression of PD [4].

Other scholars assume that excitotoxicity induced by increased glutamate activates both synaptic and extrasynaptic NMDARs, triggering intracellular Ca^2+^ levels to rise, activating nNOS [44]. Lastly, NO stimulates its own production by Ca^2+^ release into the cytoplasm from the endoplasmic reticulum, the mitochondria, or through activation of the NMDAR [45].

## 4. Role of NO in PD

NO is a gaseous molecule implicated in a wide range of physiological processes, such as vascular tone regulation, muscular contraction/relaxation, platelet aggregation, neurodegeneration, and neurotransmission [46]; however, depending on the intracellular redox state and its concentration, it can exert a neurotoxic action, thus causing neurodegenerative disorders such as PD.

**Figure 2:**
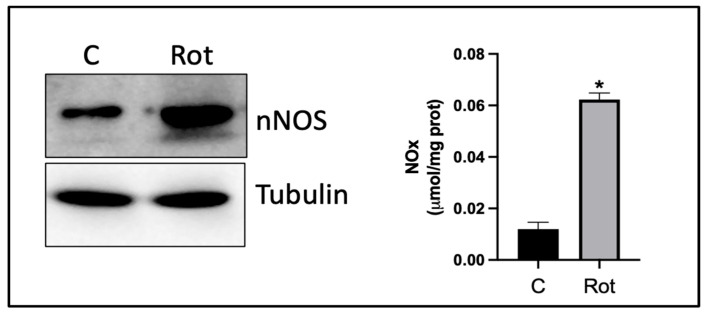
nNOS content and activity in SH-SY5Y neuroblastoma cells after rotenone treatment. SH-SY5Y cells were treated with 5 mM rotenone. After 24 h, nNOS content and NO levels were evaluated by Western blot analysis and Griess reaction. Then, 10 g of total protein extracts were loaded for detection of nNOS protein content by Western blot analysis. a-Tubulin was used as loading control. Immunoblots are from one experiment representative of six that gave similar results. To determine the activity of nNOS, the Griess reaction method was used to measure the total amount of nitrites plus nitrates (NOx) released in the culture medium. Data are reported as mmol/mg prot and expressed as means ± S.D. (*n* = 3; * *p* < 0.001).

In more detail, NO plays a crucial role in synaptic transmission, acting as a retrograde signaling molecule in the central nervous system. Specifically, NO produced by nNOS in postsynaptic neurons diffuses back to presynaptic neurons. This retrograde signaling influences neurotransmitter release and is essential for processes such as long-term potentiation (LTP), a key mechanism underlying learning and memory. In regions such as the hippocampus and the anterior cingulate cortex, NO modulates LTP through pathways involving NMDA receptors and calcium signaling [47]. Once released, NO activates soluble guanylyl cyclase (sGC) in presynaptic terminals, leading to the production of cGMP. This cascade facilitates neurotransmitter release and increases synaptic strength. The effects of NO are pathway-specific, meaning that its influence varies depending on the type of synapse and the brain region [48]. Thus, dysregulation of NO signaling has been implicated in conditions such as neurodegeneration and PD, where excessive NO may contribute to oxidative stress and synaptic dysfunction. In support of this, unpublished data from our laboratory demonstrate increased NO production in SH-SY5Y dopaminergic cells treated with rotenone, a chemical compound that plays a significant role in PD research because it is used as an experimental model to induce Parkinson’s disease (PD)-like symptoms and pathologies in animal and cellular models (Figure 2). Furthermore, these data also show increased nNOS protein levels compared to untreated cells, confirming the negative role that NO may play in disease progression.

NO plays an inhibitory role in non-cholinergic noradrenergic (NANC) transmission in the peripheral nervous system, a phenomenon known as nitrergic neurotransmission [47]. This process interacts with noradrenergic and cholinergic pathways, modulating their activity to regulate the conduction of action potentials. Depending on both context and physiological requirements, NO can enhance or inhibit these pathways, demonstrating a dual regulatory role that balances excitation and inhibition [47]. Adequate NO synthesis plays a crucial role in memory formation. Due to its gaseous nature and high diffusion capacity, NO can move from the postsynaptic to the presynaptic terminal, autonomously stimulating the release of neurotransmitters. This process gives rise to the LTP activation cycle, considered the physiological mechanism underlying learning and memory [49]. The widespread presence of NMDAR in the CNS underscores their relevance, as well as that of NO, in synaptic modulation and in learning and memory processes [50]. The half-life of NO is influenced by its concentration and the local redox environment, mainly through second-order reactions either with oxygen to form nitrogen dioxide (NO_2_) or with superoxide to form peroxynitrite (ONOO^−^). Under normal physiological conditions, NO has a half-life on the order of seconds, but this can be significantly reduced in pathological states where oxidative stress increases superoxide availability. For example, in the presence of higher NO concentrations associated with inflammation or neurodegeneration, the half-life may decrease, accelerating the formation of reactive nitrogen species (RNS) and altering the balance between the protective and toxic effects of NO. The production of NO can be experimentally regulated or inhibited by N-monomethyl-L-arginine (L-NMMA), a competitive inhibitor of NOS commonly used in research. Endogenous concentrations of L-NMMA, particularly in vessels, are considered low. In contrast, asymmetric dimethylarginine (ADMA), another endogenous NOS inhibitor, is more prevalent, especially in cardiovascular and neurodegenerative conditions. While ADMA is well characterized in vessels, its role in the nervous system remains less explored, but could be significant in modulating NO production in pathological conditions [50]. At the neuronal level, NO produced along synaptic spines can easily react with sGC, leading to the synthesis of cyclic guanosine 3′5′-monophosphate (cGMP). In this way, cGMP regulates neuronal dynamics, growth, and synaptic plasticity, also through the modulation of cGMP-dependent kinase (cGKI or PKG), cyclic nucleotide-gated ion channels (CNG), and cGMP-dependent nucleotide phosphodiesterases.

NO through protein modifications, such as nitrosylation and nitration, controls synaptic activity. Protein nitration typically adds a nitro group (–NO2) to one of the two carbon atoms in position 3 of the aromatic ring of tyrosine residues to form nitrotyrosine. An increase in nitrotyrosine has been detected in the *substantia nigra* in in vivo models of PD [51]. Under homeostatic conditions, NO protects neurons from hyperexcitability by S-nitrosylation (covalent attachment of a NO group to cysteine residues on proteins) and subsequent inhibition of NMDAR (SNO-NMDARs) [52]. In addition to NMDAR regulation, NO can also protect neurons from hyperexcitability by modulating the expression of alpha-amino-3-hydroxy-5-methyl-4-isoxazole (AMPA) receptors (AMPARs) through their S-nitrosylation [53]. Furthermore, NO can function as a neurotransmitter, regulating learning and memory formation, Ca^2+^ signaling, and stimulating extracellular vesicle release and endocytosis [54]. In contrast, under conditions of high NO production and/or nitrosative stress, as in the specific case of PD neurons, NO impairs axodendritic function by compromising synaptic signaling, vesicular trafficking, and dopamine homeostasis, and may cause DNA damage [7].

As for S-nitrosylation of synaptic proteins by NO, it has been clarified that S-nitrosylating protein disulfide isomerase or microtubule-associated protein 1b impairs neurite length [55,56]. Such axodendritic impairments cause cognitive decline and neuronal death due to loss of network connectivity. NO regulates NMDAR-mediated excitotoxicity, a phenomenon that can be enhanced by molecules such as SNO-Src and SNO-SHP-2 [57,58]. Src, a protein tyrosine kinase, can be activated by autophosphorylation and S-nitrosylation, which leads to phosphorylation of the NR2B subunit of the NMDA receptor, thereby increasing its activity. Additionally, SHP-2, a phosphatase that interacts with Src, is involved in cell survival through the ERK1/2 signaling pathway, but its function is inhibited by nitrosylation. Ca^2+^ influx through the NMDAR activates nNOS, which synthesizes NO, thus creating a cycle in which excess NO further increases the activity of SNO-Src and SNO-SHP-2, intensifying excitotoxicity [59]. This mechanism leads to continued production of NO, which, in patients with PD, may contribute to a vicious cycle that further damages nerve cells. This complex mechanism highlights how disruption of NO-mediated signaling systems may play a significant role in pathological processes like PD, contributing to the neurodegeneration and motor difficulties associated with the disease. At the neuronal level, NO is also able to regulate the intracellular traffic of vesicles and cargo. More specifically, it has been demonstrated that the use of agrochemicals in post aminergic neurons of PD patients caused the nitration of tubulin, preventing the transport of the kinesin superfamily protein (KIF5b, KIF5a and KIF21B) towards the mitochondria, determining an energy deficit at the synapse, as well as a reduced production of neurotransmitters [60]. This phenomenon would seem to be amplified by the S-nitrosylation of the vesicular monoamine transporter (VMAT2), which is implicated in the transport of important enzymes responsible for the synthesis of dopamine [61].

Another protein found S-nitrosylated in PD is protein disulfide isomerase (PDI), which catalyzes thiol-disulfide exchange in the endoplasmic reticulum (ER), thereby facilitating disulfide bond formation and rearrangement reactions. When PDI is S-nitrosylated, it is inhibited; this leads to an accumulation of polyubiquitinated proteins, leading to neuronal cell death [62]. Many other S-nitrosylations of key proteins in the pathogenesis of PD have also been identified, such as DJ-1, PINK, and Parkin.

**Figure 3:**
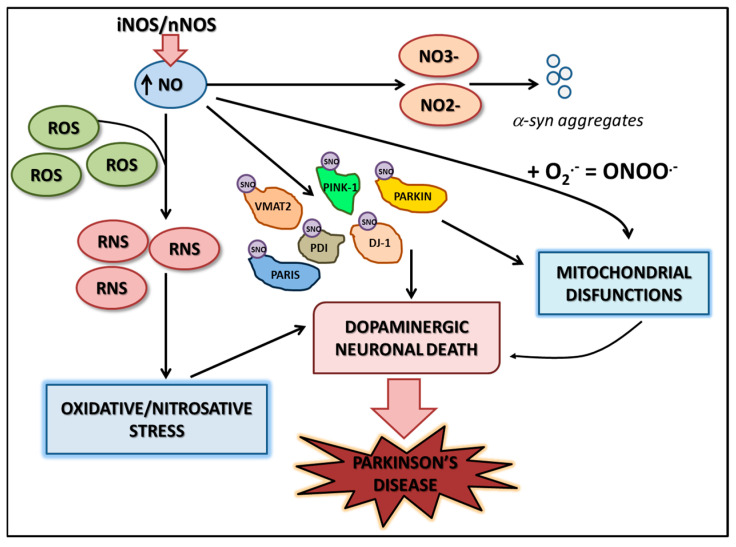
High concentrations of NO can induce negative effects on neuronal functionality either directly or through modifications at the protein level (nitrosylation and nitration). For example, S-nitrosylation of proteins involved in mitochondrial functionality and homeostasis (such as DJ-1 and PINK-1) damages their integrity, putting neuronal survival at risk. S-nitrosylation of proteins involved in protein degradation (such as PDI and Parkin) causes the loss of their function and a corresponding increase in intracellular proteins with the formation of Lewy bodies. NO can also act directly by inhibiting complexes I, III, and IV at the mitochondrial level, with a concomitant reduction in ATP production, cytochrome *c* release, and apoptosis. Finally, combined with superoxide, NO leads to the formation of peroxynitrite, which damages mitochondria and mtDNA, thus inducing neuronal apoptosis.

Parkin, a ubiquitin ligase crucial for mitochondrial function, is subject to S-nitrosylation, leading to impaired mitophagy and oxidative stress. Figure 3 illustrates the key proteins affected by S-nitrosylation and nitration, highlighting their roles in mitochondrial dysfunction, oxidative stress, and neuronal death in PD.

DJ-1 has a protective role, limits cell death, and promotes survival. Thus, its nitrosylation (on some conserved cysteines) would lead to loss of function, mitochondrial dysfunction, oxidative stress, protein aggregation, and autophagy defects [63]. Parkin is a ubiquitin involved in protein degradation, mitochondrial function, and endoplasmic reticulum (ER) stress [64]. Parkin also regulates mitochondrial function through mitophagy, thus limiting ROS formation [65]. Several studies suggested a possible implication of damaged Parkin in PD pathophysiology. Recent scientific research has shown the presence of SNO-Parkin in PD patients and mice treated with rotenone and MPTP, and an increased depolarization of the inner mitochondrial membrane when SNO-Parkin levels were high [66]. S-nitrosylation of Parkin determines the loss of its functionality accompanied by oxidative stress, the accumulation of protein aggregates, endoplasmic reticulum stress, and neuronal death by apoptosis [67]. PINK1 is a serine/threonine kinase involved in mitochondrial homeostasis and mitophagy, as well as in the inhibition of ROS-induced apoptosis [68]. Yet, it collaborates with Parkin in the mechanisms of mitochondrial fusion and fission [69]. It has been proven that the loss of function in PINK1 due to S-nitrosylation (C568 was identified as the most likely site) leads to an impairment of mitophagy, a neuronal death in in vitro models of PD, thus establishing a basis for PD pathogenesis [70].

Another protein found to be S-nitrosylated in mouse models of PD is Parkin-interacting substrate (PARIS), a transcriptional repressor of peroxisome proliferator-activated receptor-gamma coactivator alpha (PGC-1α). In these mouse models, besides an accumulation of PARIS and SNO-PARIS, an insoluble sequestration of PGC-1α in *substantia nigra* occurs, resulting in a reduction in the mtDNA copy number and ATP concentration that were restored by N(ω)-nitro-L-arginine methyl ester, a NOS inhibitor [71]. These results suggest that modulation of NO can be a therapeutic for SNO-PARIS-mediated neurodegeneration.

The reaction between NO and superoxide leads to the formation of peroxynitrite (ONOO^−^), a process accelerated in PD due to the increased production of superoxide due to dopamine metabolism [67]. ONOO^−^ is considered one of the most damaging molecules in the progression of PD for its ability to bind to mitochondrial complexes (I, III, IV), mitochondrial DNA, and nuclear DNA, causing mitochondrial apoptosis, necrosis, and autophagy [72]. In addition, ONOO^−^ reduces dopamine transport in dopaminergic neurons through the inhibition of the presynaptic dopamine transporter [73]. NO also contributes to the release of iron from transferrin, promoting the generation of ROS and triggering ferroptosis, a specific type of cell death mediated by iron accumulation [74]. Excess NO and ONOO^−^ can damage DNA directly or by hindering DNA synthesis and repair mechanisms, resulting in apoptotic processes [74].

Dopamine and its metabolites may play a central role in the pathogenesis of PD, allowing us to better understand why dopaminergic neurons are particularly vulnerable in this condition. Importantly, both tyrosine hydroxylase, an essential enzyme in dopamine synthesis, and NOS are present in dopaminergic neurons [18]. There are multiple interactions between NO and dopamine metabolism, as demonstrated by the possible reduction in available dopamine due to increased levels of NO and ONOO^−^. Oxidation of dopamine leads to the formation of dopamine quinone, a toxic molecule that interferes with mitochondrial function, causing a reduction in ATP and glutathione levels, as well as an increase in ROS [18,75]. Another byproduct of dopamine metabolism, 3,4-dihydroxyphenylacetic acid (DOPAC), is formed in the mitochondrial matrix and its oxidation by NO (produced by mtNOS) impairs the activity of cytochrome c oxidase (complex IV), further damaging mitochondria. In addition, the interaction between DOPAC and NO appears to act synergistically, contributing to a reduction in glutathione levels and thus increasing oxidative stress [72]. Finally, the superoxide anion generated by dopamine metabolism binds to NO to produce ONOO^−^,which induces DNA damage, protein misfolding, mitochondrial dysfunction, and, ultimately, cell death [67,72].

Other neurotoxic effects of NO include base deamination, which induces DNA damage, activation of the enzyme poly(ADPribose)-synthase, and depletion of ATP and nicotinamide adenine dinucleotide (NAD), which leads to cell death. In addition, RNS have been shown to inhibit ribonucleotide reductase, blocking the synthesis of new DNA and causing DNA single-strand breaks. These events trigger signaling pathways such as p53, which activates nuclear poly (ADP-ribose) polymerase (PARP-1), which causes apoptosis in mouse models of PD [76]. Its high affinity for heme is another factor that could explain NO toxicity. Inhibition of cytochrome *c* oxidase activity makes NO a physiological regulator of mitochondrial electron transfer and ATP synthesis [77]. ATP synthesis is also impaired by nitrosylation or oxidation of protein thiols and the subsequent removal of iron from iron–sulfur clusters in cytochrome complexes by NO [78]. Furthermore, there is a possibility that NO is the main element responsible for the preferential inhibition of complex I in dopaminergic neurons in PD [79].

## 5. Potential Role of Dietary Nitrate in Parkinson’s Disease

### 5.1. Nitrate Metabolism

Despite the limited clinical evidence directly linking dietary nitrate to PD, this section, moving from preclinical studies and the known mechanisms of the nitrate–nitrite–NO pathway, aims at exploring the potential benefits of nitrate. The human body receives nitrate through diet and endogenous formation. In the second case, nitrate and nitrite are produced in the L-arginine–NO-synthase pathway. NO is formed within endothelial cells, where the metabolism of L-arginine, catalyzed by NOS, leads to the formation of L-citrulline and NO [14]. NO so formed is highly reactive; an excess amount is rapidly oxidized in the blood by oxyheme proteins (oxyhemoglobin or oxymyoglobin) into nitrites and nitrates [80]. However, the formation of endogenous NO by this pathway is inhibited under conditions of hypoxia or ischemia [81]. Thus, the main pathway of intake appears to be through food. The nitrate–nitrite–NO pathway, as first described in 2008 by Lundberg et al., encompasses the entero-salivary circle as a critical component of nitrate metabolism and NO production [14]. This pathway provides a mechanism for NO generation which is independent of the enzymatic activity of nitric oxide synthases, making it particularly relevant under conditions of hypoxia or ischemia. Dietary nitrate is partially reduced to nitrite and thus to biologically active nitrogen oxides, which perform various physiological functions [82]. A significant portion of dietary nitrate is reduced to nitrite in the oral cavity by commensal bacteria, with a typical salivary nitrate-to-nitrite ratio of approximately 2:1 to 3:1, corresponding to 25–33% [83]. Recent studies confirm this conversion, highlighting how the abundance of nitrate-reducing bacteria in the oropharynx significantly influences salivary nitrite production and systemic NO levels [84]. Another study demonstrated that *Veillonella* spp. and *Prevotella* spp. are the predominant bacteria involved in the reduction from nitrate to nitrite in saliva [85]. After deglutition, due to the acidic gastric environment, nitrite is reduced to NO and biologically active nitrogen oxides, such as NO_2_ and N_2_O_3_, through non-enzymatic processes. Nitrate reduction primarily occurs in the oral cavity via lingual bacterial nitrate reductases, with minor contributions from xanthine oxidoreductase (XOR) in the liver [84]. Nitrite reduction in the stomach may be supported by nutrients such as ascorbic acid and polyphenols [86]. In this process, commensal bacteria play a crucial role in the reduction from nitrate to nitrite, highlighting the critical interaction between diet and microbiota. Recent studies emphasize the need for up-to-date estimates of the nitrate–nitrite conversion ratio, considering variables such as individual microbiota composition, dietary patterns, and oral hygiene practices. This is particularly important in conditions where NO bioavailability may influence neurovascular health and disease progression, including PD [87]. In the small intestine, however, NO is absorbed into the bloodstream. NO present in blood and tissues can undergo spontaneous oxidation to nitrite and nitrate. Excess nitrate is excreted via the urine (approximately 75% of the total nitrate), while the remainder is reabsorbed and concentrated in the salivary glands and then secreted into the saliva [88]. Under acidic conditions, nitrate remains highly stable. Nitrite, however, undergoes disproportionation at low pH, producing nitric oxide (NO), nitrogen dioxide (NO_2_), and other reactive nitrogen species. This process can be further influenced by the presence of reducing agents such as ascorbic acid and polyphenols. In the entire gastrointestinal tract, nitrate can react with amines and amides to the formation of N-nitroso [82]. Reactions between primary amines and nitrite form unstable nitrosamines, which are degraded to nitrogen and alcohol [82]. Some nitrate is then excreted with the urine in the form of urea and ammonia. Factors affecting nitrate metabolism are gastric pH, iron, polyphenols, and vitamins C and E [89,90]. Dietary nitrate, found mainly in vegetables such as beetroot and spinach, increases the bioavailability of NO via the nitrate–nitrite–NO pathway. This mechanism could potentially counteract the main pathological features of PD, such as neuroinflammation and oxidative stress. However, current evidence is largely pre-clinical and its relevance for PD patients has yet to be confirmed.

### 5.2. Food Sources a Potential Dietary Strategy to Increase NO Bioavailability

Nitrate and nitrite are natural compounds found in vegetables, water, and processed meats. Dietary nitrate, predominantly found in vegetables such as beets, spinach, and other leafy greens, represents a promising non-pharmacological strategy to enhance NO bioavailability. This is particularly relevant in the context of PD, where NO plays a crucial role in neuronal health, vascular function, and oxidative stress management. The nitrate–nitrite–NO pathway provides an alternative mechanism for NO production, independent of eNOS, which can be particularly beneficial in neurodegenerative diseases where endothelial dysfunction is common. Studies indicate that nitrate-rich diets may support vascular health, improve mitochondrial efficiency, and reduce oxidative stress, all factors implicated in PD pathology. Leveraging nitrate-rich vegetables as a dietary intervention may thus offer a dual advantage of neuroprotection and vascular support, providing a natural and accessible adjunct to conventional PD treatments. Increased NO bioavailability from dietary sources improves cerebral blood flow, reduces oxidative stress, and supports mitochondrial function, all of which are critical in mitigating PD pathology [91]. For example, regular consumption of 300–400 g of nitrate-rich vegetables or 500 mL of beetroot juice has been shown to significantly elevate plasma nitrate levels, enhancing neurovascular health [19].

The main sources of dietary nitrate intake are vegetables and fruit, which contribute up to 75% of the total dietary intake [15]. Factors influencing nitrate accumulation in plants are diverse and include plant type, cultivation techniques, and post-harvest treatment. Studies have shown that leafy vegetables tend to have higher nitrate levels than seeds or tubers. Thus, rocket, lettuce, and spinach have higher nitrate contents. Beetroot and celery also contain a significant amount of nitrate [82]. Studies report that the nitrate content varies in relation to the anatomical parts of the plant. The parts of the plant that contain the most nitrate can be classified as follows: petiole, leaf, stem, root, inflorescence, tuber, bulb, fruit, and seed [92]. NO levels have been shown to decrease during fruit ripening [93,94]. Thus, a diet that favors the leaves or stems of the plant allows a higher NO intake.

Agronomic practices play an important role in the accumulation of NO in the plant. Fertilizer use, soil conditions, growth rate, and growing conditions, including light intensity and rainfall level, significantly influence nitrate content in vegetables [15]. Seasonality therefore influences nitrate content. Studies have shown that post-harvest treatments, such as heat treatments and storage conditions, can also affect the reduction in nitrate content [82]. Increasing the temperature during storage of vegetables influences the decrease in nitrite content, due to the increase in the bacterial-facilitated reduction from nitrate to nitrite [95]. Processes such as acidification, brining, and pasteurization also reduce the nitrate content compared to fresh counterparts [96]. These studies allow us to infer that consuming fresh vegetables allows a higher intake of NO, compared to the same processed or out-of-season products.

Nitrate can also be ingested through other foods, such as water and meat products. Water accounts for 20% of the daily intake of nitrate and nitrite, and meat products for 6% [15,97]. The meat industry uses nitrate/nitrite as additives in the meat curing process. In these products, the formation of nitric oxide determines the subsequent decrease in the amount of these compounds following reactions with myoglobin and other substrates, including amino acids such as cysteine [15,98]. Only 10–20% of the originally added nitrite is present in meat products after production, and this residual amount slowly decreases during the storage period [15]. Studies are underway to optimize the use of ‘natural’ sources of nitrate as additives, such as celery juice and beet or spinach extract [99]. Several studies have shown that it is feasible and potentially useful to use natural alternatives to nitrate and nitrite in meat products [100,101,102]. In addition to water and meat products, nitrate can be ingested through nitrate-rich vegetables, which are key dietary sources for improving NO bioavailability. Table 1 summarizes the nitrate content of commonly consumed vegetables, recommended portion sizes, and references supporting these values, providing practical guidance for dietary strategies.

The intakes presented in Table 1 derive from a combination of clinical studies, observational data, and experimental evidence. While some sources, e.g., beetroot and spinach, are supported by robust clinical studies examining nitrate intake thresholds for physiological effects, others rely on estimates or less complete data sets. This variability reflects differences in study design, populations, and nitrate bioavailability between food sources. In addition, factors like agricultural practices, food preparation, and individual variability in nitrate metabolism may affect the nitrate content and efficacy of these portions. These discrepancies highlight the need for standardized guidelines based on well-designed studies to ensure consistent, evidence-based dietary recommendations.

## 6. Preclinical and Clinical Evidence

### 6.1. Preclinical Studies

Several preclinical animal models have highlighted the neuroprotective effects of dietary nitrate, demonstrating its potential in mitigating neuroinflammation, oxidative stress, and other pathological features associated with neurological disorders. The conversion of dietary nitrate to NO via the nitrate–nitrite pathway promotes vasodilation, reduces inflammation, and attenuates oxidative stress. This pathway has shown cytoprotective effects against ischemic damage and inflammatory responses in preclinical animal models, highlighting the role of nitrate/nitrite as mediators to limit oxidative injury and inflammation [108]. In animal studies, nitrate reduced leukocyte recruitment and adhesion in inflammatory responses, potentially preventing inflammatory tissue damage. This anti-inflammatory effect also resulted in a reduction in markers such as ICAM-1 and P-selectin, which are crucial for controlling inflammation [109]. Dietary nitrate reduced oxidative stress and inflammation in cardiovascular models, probably due to the effects of NO on reducing the activity of NADPH oxidase, a key enzyme that determines oxidative damage. This effect was observed in animal models of hypertension, where nitrate improved vascular health and reduced oxidative markers [106]. In studies aimed at neuroprotection, NO-releasing compounds were found to reduce neuroinflammation and neuronal damage in models of Alzheimer’s disease, indicating that dietary nitrate and NO donors might have a similar effect in reducing inflammation in brain tissue [110]. Table 2 summarizes preclinical studies highlighting the neuroprotective and cardiovascular effects of dietary nitrate. Selected studies focus on physiological mechanisms such as ischemic tolerance, reductions in oxidative stress and inflammation, and inhibition of endothelial dysfunction, demonstrating the potential of nitrate for applications in neurodegenerative and cardiovascular contexts.

Table 2 includes preclinical studies on the effects of dietary nitrate. The following acronyms were used: NOS (nitric oxide synthase), NO (nitric oxide), ICAM-1 (intercellular adhesion molecule 1), NMDA (N-methyl-D-aspartate), NADPH (nicotinamide adenine dinucleotide phosphate), and NSAID (non-steroidal anti-inflammatory drugs).

These studies highlight the potential of dietary nitrate as a therapeutic agent, particularly for neuroprotective applications, also demonstrating cardiovascular health benefits in specific preclinical models. This evidence supports further exploration of its translational value in human health contexts, building on existing research on its cardiovascular effects since 2006 [118].

### 6.2. Clinical Studies

Although no direct clinical trials or published studies have specifically examined dietary nitrates in PD treatment, their therapeutic potential has attracted increasing interest due to their role as precursors of NO, a molecule with important neurovascular, anti-inflammatory, and anti-oxidative properties. Clinical studies in related areas demonstrate the ability of dietary nitrate to increase cerebral blood flow, improve cognitive function, and reduce vascular dysfunction, all of which are relevant to the pathophysiology of PD. For instance, Presley et al., (2011) [119] showed that nitrate supplementation increases cerebral blood flow in regions critical for executive functions (see Table 3), while Aamand et al., (2013) [120] demonstrated improved neurovascular coupling and hemodynamic responses to visual stimuli via the nitrate–nitrite–NO pathway. These findings suggest potential pathways to mitigate neurovascular deficits in PD. Kapil et al., (2015) further emphasized the systemic vascular benefits of nitrate supplementation, demonstrating its ability to reduce blood pressure through NO-mediated vasodilation [121]. This is particularly relevant for patients with PD, who often present vascular dysfunction in comorbidity. Cognitive improvements associated with nitrate supplementation have also been reported. Wightman et al., (2015) observed increased cerebral blood flow and cognitive performance in healthy adults after nitrate supplementation, suggesting potential applications for addressing cognitive symptoms in PD [122]. In contrast, Molina et al., (1994) found no significant differences in plasma nitrate levels between Parkinson’s patients and healthy controls [123], which indicates that basal nitrate levels may not be directly altered in PD, emphasizing the need to evaluate functional outcomes of nitrate-derived NO rather than static concentrations.

This interest arises primarily from preclinical findings and the established role of NO in neurovascular health. However, the absence of clinical trials which specifically target dietary nitrates in Parkinson’s patients represents a critical gap in the literature, underscoring the need for rigorous studies to validate these potential therapeutic benefits. Table 3 summarizes the main clinical studies that have focused on dietary nitrate, emphasizing its broader implications for neurovascular and systemic health, and its potential relevance for PD management.

Table 3 provides an overview of clinical studies evaluating the effects of dietary nitrate in Parkinson’s disease (PD) and related conditions. The table includes study types, key findings, mechanisms explored involving nitric oxide (NO), models or systems used, and potential applications or implications. Abbreviations: PD, Parkinson’s disease; NO, nitric oxide; BOLD-fMRI, functional MRI dependent on blood oxygen level.

## 7. Future Perspectives

### 7.1. Dietary Interventions

Dietary nitrate supplementation, primarily through nitrate-rich vegetables like beets, spinach, and arugula, is emerging as a potential adjunct to standard therapies for PD. The nitrate–nitrite–NO pathway provides an alternative mechanism for generating NO, bypassing the eNOS pathway, which is often impaired in PD. This alternative pathway can enhance cerebral blood flow, reduce oxidative stress, and improve mitochondrial efficiency, which are critical in managing neurodegenerative diseases.

Research supports the idea that nitrate supplementation may complement standard dopaminergic therapies by targeting non-dopaminergic pathways, addressing neurovascular dysfunction and neuroinflammation, both of which are key contributors to disease progression. For example, studies in aging populations have shown improvements in vascular and cognitive health with nitrate-rich diets, suggesting potential benefits for PD patients [124]. Additionally, preclinical studies demonstrate that dietary nitrate reduces oxidative stress and protects mitochondrial complex I function, which may help slow neuronal degeneration [125]. The practical application involves the daily integration of 300–500 mg of dietary nitrate, achievable through the consumption of approximately 150–250 g of nitrate-rich vegetables or 500 mL of beetroot juice. The European Food Safety Authority (EFSA) has set an acceptable daily intake (ADI) for nitrate at 3.7 mg per kg of body weight. For a 70 kg adult, this translates to ~260 mg per day. However, therapeutic doses in clinical settings often exceed this limit without adverse effects when derived from natural sources [126]. However, emerging evidence highlights concerns regarding adverse effects, particularly the formation of potent carcinogens like N-nitrosamines. Nitrate, when ingested, can be reduced to nitrite by oral bacteria. Under acidic gastric conditions, nitrite may further convert into N-nitrosamines. N-nitrosamines, such as N-nitrosodimethylamine (NDMA), are classified as probable human carcinogens by the International Agency for Research on Cancer (IARC). This conversion is particularly relevant in the context of high nitrite intake from processed meats. Recent meta-analyses and systematic reviews provide robust evidence linking high nitrite and N-nitrosamine intake to an increased risk of gastrointestinal cancers [127]. Similarly, NDMA intake from processed meats has been identified as a key factor in colorectal cancer development [128]. A systematic review of nitrate and nitrite intake concluded that, while high nitrate consumption from vegetables is generally associated with reduced gastric cancer risk, elevated nitrite and NDMA levels contribute to a heightened risk [129]. The study underscores the differential impact of dietary sources, suggesting that vegetable nitrate from leafy greens and beetroot, which often contain antioxidants and other nutrients that mitigate potential risks associated with nitrate exposure, may confer protective effects, whereas processed meat-derived nitrite poses carcinogenic risks. Vegetables, rich in antioxidants, may mitigate the nitrosation process, thereby reducing the risk of N-nitrosamine formation. Conversely, processed meats lack these protective compounds, increasing the likelihood of nitrosamine-related carcinogenesis. Future research should refine dietary guidelines to minimize nitrosamine exposure while preserving the cardiovascular and neuroprotective benefits of nitrate-rich diets. Additionally, exploring alternative NO modulating therapies, e.g., curcumin and isocoumarins, could provide complementary approaches to nitrate supplementation [130,131]. These bioactive compounds, known for their anti-inflammatory and antioxidant properties, may inhibit excessive NO production and help manage neuroinflammation, broadening the scope of therapeutic avenues.

Future research should focus on combining nitrate supplementation with existing PD treatments to evaluate synergistic effects on motor symptoms, cognitive function, and disease progression. Such dietary strategies may offer a novel, non-invasive approach to complement pharmacological treatments in PD. The safety of patients with Parkinson’s disease must be guaranteed by monitoring activities and personalized dose optimization.

### 7.2. Targeted Therapies

Targeted modulation of NOS activity offers a promising therapeutic avenue for balancing the neuroprotective benefits of NO while mitigating its potential toxic effects in PD. NO plays a dual role in neurodegeneration: At physiological levels, it supports neuronal health by promoting mitochondrial function, enhancing cerebral blood flow, and reducing oxidative stress. However, excessive NO production, particularly through iNOS, can lead to the formation of RNS, which causes oxidative damage, protein nitration, and mitochondrial dysfunction, contributing to neuronal loss in PD.

Therapeutic strategies targeting NOS activity focus on selective inhibition of iNOS while preserving or enhancing eNOS and nNOS activity. This selective modulation aims to reduce pro-inflammatory and neurotoxic NO production without impairing the neurovascular and mitochondrial benefits. Preclinical studies suggest that pharmacological inhibitors of iNOS can attenuate neuroinflammation and oxidative damage in PD models, providing a neuroprotective effect [125]. Additionally, it has been proven that targeting NOS activity to restore NO balance improve mitochondrial bioenergetics and reduce ROS formation, addressing two key mechanisms underlying PD progression.

Future approaches may involve the development of highly specific NOS modulators, such as inhibitors for iNOS or activators of eNOS/nNOS, possibly combined with dietary nitrate supplementation to enhance systemic NO bioavailability safely. Gene therapy to regulate NOS isoform expression in affected brain regions is another emerging possibility. However, further research is required to optimize the therapeutic window and minimize off-target effects, particularly in maintaining the delicate balance between protective and harmful NO levels. These targeted therapies could serve as valuable adjuncts to existing PD treatments, offering a more comprehensive strategy to slow disease progression and improve patient outcomes.

### 7.3. Needed Studies

Despite encouraging preclinical and observational evidence, the role of dietary nitrate as adjunctive therapy for PD remains unexplored in the clinical setting. The limited number of clinical studies directly targeting dietary nitrate in PD represents a significant gap in the current knowledge base. Rigorous and well-designed RCTs are essential to assess their efficacy and safety, with a focus on their effects on motor and non-motor symptoms, neurovascular health, oxidative stress, and overall disease progression.

Future studies should aim to answer several key questions, including the optimal dosage, duration, and methods of administration of dietary nitrate, whether from whole foods (e.g., nitrate-rich vegetables) or concentrated supplements (e.g., beetroot juice). Research must also consider variability in disease stage, treatment regimens, and dietary habits within different PD populations. Comprehensive outcome measures, incorporating both clinical endpoints (e.g., motor function, quality of life) and biomarkers (e.g., NO bioavailability, oxidative stress markers, mitochondrial function), will be critical to provide mechanistic insights. By addressing these gaps, future research will clarify the therapeutic potential of dietary nitrate and inform their incorporation into evidence-based guidelines for the management of PD.

### 7.4. Limitations of the Studies Reviewed

The studies reviewed have several limitations. Methodological constraints, such as small-size samples, particularly in clinical studies, and dependence on preclinical models, limit the application of the results to the large population affected by Parkinson’s disease. Many studies have used animal models that may not fully replicate the complexity of human neurodegenerative processes. Furthermore, variations in study designs, such as differences in dietary nitrate dosages, duration of interventions, and a lack of standardized protocols, make direct comparisons difficult. Observational studies often suffer from confounding factors and potential biases, such as publication bias or selective reporting. Furthermore, clinical studies on dietary nitrate in Parkinson’s disease are scarce, which underlines the need for larger, well-designed randomized controlled trials to validate the preclinical evidence.

### 7.5. Comparative Analysis of Results

Despite these limitations, consistent patterns also emerged from the studies reviewed. Both preclinical and clinical studies suggest that dietary nitrate effectively increases nitric oxide bioavailability, attenuates oxidative stress, and improves neurovascular function. However, divergent results are evident. For example, preclinical models consistently report neuroprotection through a reduction in inflammation and oxidative damage, while clinical studies, as in Molina et al., (1994) [123], fail to demonstrate significant differences in plasma nitrate levels between Parkinson’s patients and group controls. In particular, clinical trials focusing on nitrate-rich diets proved promising for the improvement of cognitive and vascular outcomes, aligning with mechanistic insights from preclinical research [119,122]. Future studies should aim to fill these gaps by adopting standardized protocols and integrating preclinical findings with clinical applications.

## 8. Conclusions

NO proved pivotal in the pathophysiology of PD, oscillating between neuroprotection and neurotoxicity. NO generated by eNOS and nNOS supports vascular homeostasis, mitochondrial function, and synaptic plasticity, which are crucial in counteracting neurodegeneration. However, excessive NO production through iNOS leads to oxidative stress, neuroinflammation, and neuronal loss, perpetuating the progression of PD.

Dietary nitrate offers a promising opportunity to modulate NO levels via the nitrate–nitrite–NO pathway. Preclinical studies and early clinical evidence suggest benefits for endothelial function and motor performance. By increasing NO bioavailability and counteracting oxidative damage, dietary nitrate may complement existing therapies to address the multiple mechanisms of PD.

Although current evidence supports NO potential, restricted clinical data in PD populations limit its immediate application. More rigorous and targeted studies are needed to confirm its efficacy, establish safety profiles, and explore its integration with existing therapies. Being an accessible and low-cost intervention, dietary nitrate promises to improve the quality of life of PD patients, pending further confirmation.

## Figures and Tables

**Table 1:** Nitrate-rich vegetables, their estimated nitrate content, and recommended serving sizes for enhancing NO bioavailability.

References	Potential Serving Size for Benefit	Nitrate Content (mg/100 g)	Food Source
[19]	~150–200 g (1–2 beets)	250–400 mg	Beetroot (raw/cooked)
[91]	~100–150 g (1 bowl of salad)	300–400 mg	Spinach (raw)
[103]	~80–100 g (1 large handful)	>400 mg	Rocket (arugula)
[104]	~150 g (1 small head)	~150 mg	Lettuce (Romaine)
[105]	~200 g (2 medium stalks)	150–200 mg	Celery (raw)
[106]	~150–200 g (6–8 radishes)	~100 mg	Radish (raw)
[107]	~300–500 mL (1–2 cups)	250–300 mg/100 mL	Beetroot juice

**Table 2:** Summary of preclinical studies on the neuroprotective and cardiovascular effect of dietary nitrate.

Applications/Implications	Model/System Used	Mechanisms Explored	Key Findings	Study Type	Year	Authors
Neuroprotection in neonatal hypoxic conditions	Neonatal ischemic model	NO-mediated ischemic tolerance	NO contributes to ischemic tolerance, reducing brain damage from hypoxic events in neonatal rats.	Animal Study	1999	Gidday et al. [111]
Potential renal and cardiac protection	Animal model (hypertension)	Oxidative stress reduction through nitrate	Nitrate lowers oxidative stress	Animal Study	2011	Carlström et al. [106]
Microvascular anti-inflammatory use	Animal model (microvascular inflammation)	Inflammation reduction via nitrate	Nitrate reduces inflammation in NSAID-induced injury.	Experimental	2012	Jädert et al. [109]
Potential diabetes management	Animal model (diabetes)	Increased NO bioavailability	Dietary nitrate enhances glucose tolerance and lipid profile in diabetic rats	Animal Study	2015	Khalifi et al. [112]
Study on safety of supplementation	Animal model	Nitrate effects on health markers	Long-term nitrate supplementation shows minimal adverse effects	Animal Study	2015	Hezel et al. [113]
Neuroprotective in neonatal hypoxic conditions	Animal model (hypoxic ischemia)	NOS inhibition in hypoxic conditions	NOS inhibition is neuroprotective in hypoxic-ischemic conditions.	Animal Study	2018	Favié et al. [114]
Hypertension control	Animal model (hypertension)	Sympathetic overactivity reduction	Nitrate reduces blood pressure through decreased sympathetic activity	Animal Study	2019	Guimarães et al. [115]
Cardioprotection	Animal model (atherosclerosis)	NADPH oxidase inhibition	Nitrate reduces endothelial dysfunction and atherosclerosis	Animal Study	2020	Peng et al. [116]
Neuroprotection against prion disease	Animal model (prion disease)	Inhibition of NO glycation pathway	NO inhibition prevents glycation and neurodegeneration in prion models.	Experimental	2021	Bourgognon et al. [117]

**Table 3:** Clinical studies investigating the role of dietary nitrate in PD and related disorders.

Applications/Implications	Model/System Used	Mechanisms Explored	Key Findings	Study Type	Year	Authors
Nitrate levels are not indicative of PD risk or progression.	Plasma and cerebrospinal fluid nitrate measurement.	Nitric oxide’s role in neurodegeneration.	No significant difference in plasma nitrate levels between PD patients and controls.	Clinical observation	1994	Molina et al. [123]
Nitrate-rich diets could improve brain health in aging populations.	Arterial spin labeling MRI in older adults.	Nitric oxide-related enhancement of blood flow in hypoxic regions.	High nitrate diets enhanced regional cerebral perfusion in areas critical for executive functioning in older adults.	Clinical study	2011	Presley et al. [119]
Potential use in enhancing neurovascular health.	Cerebral blood flow imaging with BOLD-fMRI.	Nitrate–nitrite–NO pathway in neurovascular coupling.	Dietary nitrate decreased hemodynamic lag and amplitude in the visual cortex, suggesting enhanced neurovascular coupling.	Crossover study	2013	Aamand et al. [120]
Potential for dietary nitrate as an adjunct in managing hypertension.	Human clinical trial with hypertensive patients.	Nitrate-to-nitrite conversion and its impact on vasodilation.	Dietary nitrate significantly reduced blood pressure in hypertensive patients over a 4-week period.	Double-blind clinical trial	2015	Kapil et. al. [121]
Dietary nitrate could enhance cognitive and neurovascular health.	Randomized controlled trial with healthy adults.	Increased nitric oxide availability and modulation of cerebral blood flow.	Dietary nitrate improved cerebral blood flow and cognitive performance in healthy adults.	Double-blind crossover study	2015	Wightman et al. [122]

## Data Availability

No data were generated or analyzed in this study, as it is a review of existing literature.

## Abbreviations

a-Syn, alpha-Synuclein; 6-OHDA, 6-hydroxydopamine; AMPA, alpha-amino-3-hydroxy-5-methyl-4-isoxazole; AMPARs, alpha-amino-3-hydroxy-5-methyl-4-isoxazole receptor; ATP, adenosine triphosphate; Ca^2+^, calcium; cGMP, cyclic guanosine monophosphate; CNG, cyclic nucleotide-gated ion channels; CNS, central nervous system; DOPAC, 3,4-dihydroxyphenylacetic acid; eNOS, endothelial nitric oxide synthase; ER, endoplasmic reticulum; ICAM-1, intercellular adhesion molecule 1; iNOS, inducible nitric oxide synthase; L-NMMA, N-monomethyl-L-arginine; LTP, long-term potentiation; MPTP, 1-methyl-4-phenyl-1,2,3,6-tetrahydropyridine; NAD, nicotinamide adenine dinucleotide; NADPH, nicotinamide adenine dinucleotide phosphate; NANC, non-cholinergic noradrenergic transmission; NMDAR, N-methyl-D-aspartate (NMDA) receptor; nNOS, neuronal nitric oxide synthase; NO, nitric oxide; -NO2, nitro group; NSAID, non-steroidal anti-inflammatory drugs; ONOO^−^, peroxynitrite; PARIS, Parkin-interacting substrate; PARP-1, poly (ADP-ribose) polymerase; PD, Parkinson’s disease; PDI, protein disulfide isomerase; PGC-1a, peroxisome proliferator-activated receptor-gamma coactivator alpha; RNS, reactive nitrogen species; ROS, reactive oxygen species; sGC, soluble guanylyl cyclase; SNO, S-nitrosothiol; VMAT2, vesicular monoamine transporter.
